# *MtWOX2* and *MtWOX9-1* Effects on the Embryogenic Callus Transcriptome in *Medicago truncatula*

**DOI:** 10.3390/plants13010102

**Published:** 2023-12-28

**Authors:** Elizaveta Y. Krasnoperova, Varvara E. Tvorogova, Kirill V. Smirnov, Elena P. Efremova, Elina A. Potsenkovskaia, Anastasia M. Artemiuk, Zakhar S. Konstantinov, Veronika Y. Simonova, Anna V. Brynchikova, Daria V. Yakovleva, Daria B. Pavlova, Ludmila A. Lutova

**Affiliations:** 1 Department of Genetics and Biotechnology, Saint Petersburg State University, 7/9 Universitetskaya Emb, 199034 St. Petersburg, Russia; stillinlabyrinthofmiseries@gmail.com (E.Y.K.); efremova.bio@gmail.com (E.P.E.); potsenkovskaya.ea@talantiuspeh.ru (E.A.P.); nastyaart2004@gmail.com (A.M.A.); enginequs@gmail.com (D.V.Y.); db_pavlova@mail.ru (D.B.P.); la.lutova@gmail.com (L.A.L.); 2 Plant Biology and Biotechnology Department, Sirius University of Science and Technology, 1 Olympic Avenue, 354340 Sochi, Russia; zakhar.konstantinov25@gmail.com (Z.S.K.); nikasimonova14@gmail.com (V.Y.S.); annbv19@gmail.com (A.V.B.); 3 Center for Genetic Technologies, N. I. Vavilov All-Russian Institute of Plant Genetic Resources (VIR), 42 Bolshaya Morskaya Street, 190000 St. Petersburg, Russia; 4 All-Russia Research Institute for Agricultural Microbiology, Podbelsky Chausse 3, Pushkin, 196608 St. Petersburg, Russia; kirill.vad.smirnov@gmail.com

**Keywords:** transcription factors, WOX, callus, somatic embryogenesis

## Abstract

WOX family transcription factors are well-known regulators of plant development, controlling cell proliferation and differentiation in diverse organs and tissues. Several *WOX* genes have been shown to participate in regeneration processes which take place in plant cell cultures in vitro, but the effects of most of them on tissue culture development have not been discovered yet. In this study, we evaluated the effects of *MtWOX2* gene overexpression on the embryogenic callus development and transcriptomic state in *Medicago truncatula*. According to our results, overexpression of *MtWOX2* leads to an increase in callus weight. Furthermore, transcriptomic changes in *MtWOX2* overexpressing calli are, to a large extent, opposite to the changes caused by overexpression of *MtWOX9-1*, a somatic embryogenesis stimulator. These results add new information about the mechanisms of interaction between different *WOX* genes and can be useful for the search of new regeneration regulators.

## 1. Introduction

Plant in vitro tissue cultures have a wide range of applications in biotechnology, including the obtaining of transgenic or edited plants and plant reproduction, as well as the synthesis of different biologically active compounds [1]. Despite numerous studies exploring the genetic regulation of in vitro tissue culture development, this variant of plant existence has many unknown aspects. This is probably due to the high diversity of possible cultivation conditions, multiplied by the high diversity of plant genotypes. The main processes occurring in vitro in the isolated plant tissue or organ are callus formation, i.e., unorganized cell division, and different types of regeneration, including rhizogenesis (root regeneration), caulogenesis (shoot regeneration) and somatic embryogenesis (SE), the formation of embryo-like structures which are able to develop into a new plant [2]. All these regeneration variants can be direct, when new differentiated structures are formed from the explant tissue itself, or indirect, when these structures are developed from a callus [2]. Indirect SE serves as a valuable model for plant development studies, as in this case both unorganized cell division in a callus and highly organized divisions in somatic embryos occur side by side.

*WUSCHEL-related homeobox* (*WOX*) genes, well-known regulators of cell division in plants, have demonstrated their involvement in callus formation and embryogenesis. Among these, *WOX2*, *WOX8*, and *WOX9* genes in *Arabidopsis thaliana* and their orthologs in other species play specific roles in the zygotic embryogenesis [3].

*WOX2* and *WOX8* expression begins in the zygote, while *WOX9* expression is initiated during the two-cell stage [4,5]. WOX8 and WOX9, being close relatives, play crucial roles in maintaining embryo patterning. Loss of their function leads to patterning defects in the suspensor, resulting in death [4,6]. Furthermore, expression of *WOX9* homologs is associated with SE in *Gossypium hirsutum* [7], *Phoebe bournei* [8], Hybrid Sweetgum (*Liquidambar styraciflua* × *Liquidambar formosana*) [9], *Dimocarpus longan* [10], and *Medicago truncatula* [11].

*WOX2* is specifically expressed in the apical domain of developing zygotic embryos, and loss of its function leads to the impaired control of cell division in the apical domain and increased frequency of cotyledon development disturbance. According to the genetic analysis, this function is partly carried by other *WOX* genes, including *WOX1*, *3*, and *5*, but *WOX2* plays a key role in it [6,12].

*WOX2* orthologs are also specifically expressed in somatic embryos in many plant species, serving as markers of the SE process. For instance, a high level of expression of *PpWOX2* in *Pinus pinaster* is detected in early SE. Later on, its expression decreases during maturation and development of somatic embryos [13]. High levels of expression of *WOX2* or its orthologs are associated with the early stages of somatic embryo development in other species, including *Picea abies* [14], *Dimocarpus longan* [10], Hybrid sweetgum (*Liquidambar styraciflua* × *L. formosana*) [15], *Cunninghamia lanceolata* [16], and *A. thaliana* [17,18].

WOX2 and WOX8/9 functions in zygotic or somatic embryogenesis have been shown to be interconnected in many studies. For example, WOX8 or 9 activity is necessary for maintaining proper *WOX2* expression patterns in the apical part of the zygotic embryo [6]. A recent study also showed that both *WOX2* and *8* expression patterns are disturbed in the embryos with mutations in the *YODA* gene, encoding embryo-specific protein kinase, and/or *HEAT SHOCK PROTEIN90* genes encoding YODA partners, which again confirms the connection between the *WOX8* and *2* genes [19]. In tobacco cell cultures, ectopic expression of *A. thaliana WOX2* and *WOX8* or *WOX2* and *WOX9* genes stimulated regeneration, whereas neither of these genes could induce this process on their own [20].

At the same time, the *WOX2* and *WOX8* genes may be considered as antagonists from some perspectives. Indeed, they exhibit complementary expression patterns after the first zygote division when *WOX2* is expressed in the apical cell and its descendants, whereas *WOX8* is expressed in the basal domain of the embryo [4]. Although either WOX8 or 9 induces *WOX2* expression in the apical domain [6], WOX2 is suggested to repress *WOX8* expression [21], providing a negative regulatory loop for proper embryo domain specification.

According to our previous research, MtWOX9-1, a close WOX9 relative, stimulates SE in *Medicago truncatula* [22]. The *MtWUSCHEL* gene and *STENOFOLIA* (*STF*, *MtWOX1* gene), members of the modern clade of the WOX family, can also stimulate SE in this species [23,24]. However, the effects of the *M. truncatula* WOX2 ortholog—assumed to be embryo-specific—on the in vitro regeneration and SE, have not been studied yet. To investigate the contribution of *MtWOX2* on SE in *M. truncatula*, we evaluated the effect of its overexpression on the development and transcriptome state of the embryogenic callus.

## 2. Results

### 2.1. MtWOX2 Expression Pattern during Somatic Embryogenesis

To find out whether changes occur in the *MtWOX2* gene expression level during SE, we analyzed its expression dynamics in the calli of the embryogenic line 2HA [25] and the non-embryogenic line A17 at different time points after the start of cultivation (at 0, 1, 2, 3, 4, 5 and 6 weeks) using qPCR. Contrary to our expectations, no specific expression of *MtWOX2* in the calli of the embryogenic or non-embryogenic line at any analyzed stage was detected (Figure S1).

### 2.2. MtWOX2 Overexpression Increases Callus Weight in M. truncatula

To analyze the role of *MtWOX2* in SE, we examined the SE capacity of calli overexpressing this gene. For that purpose, we transformed R108 plants with constructs for overexpression of *MtWOX2* or the beta-glucuronidase (*GUS*) gene (as a control). We cultivated transformed leaf explants on a callus induction medium containing auxin and cytokinin, and then on a hormone-free medium for regeneration. After 43 days of cultivation (including 33 days of cultivation on the callus-inducing medium and 10 days of cultivation on a hormone-free medium), samples were taken from several calli for *MtWOX2* expression analysis. According to the qPCR, calli transformed with the construct for *MtWOX2* overexpression demonstrated increased levels of *MtWOX2* transcripts (Figure S2A).

**Figure 1:**
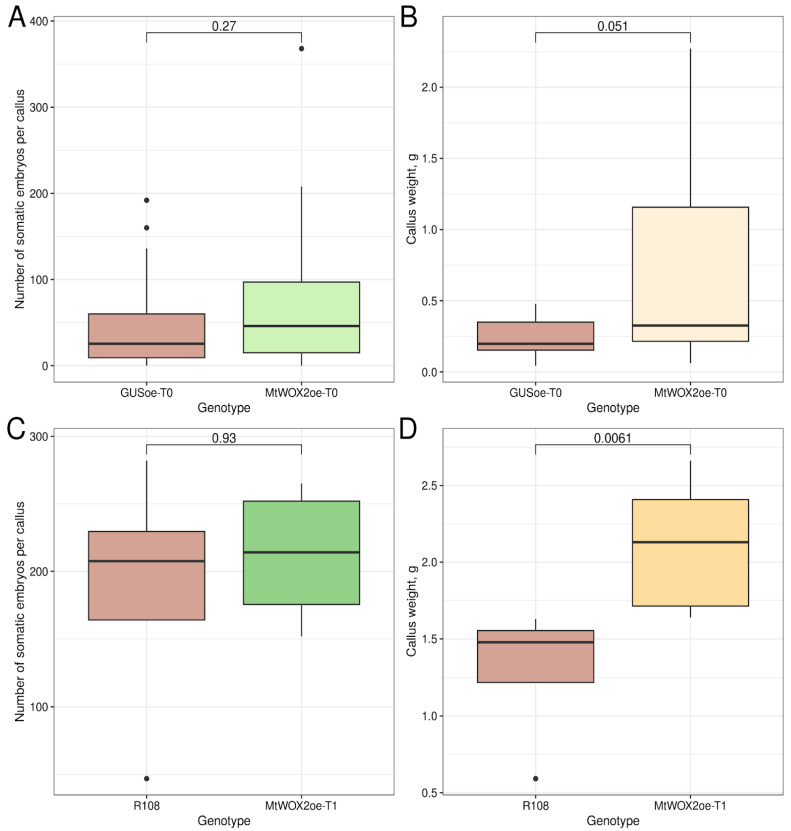
Effect of transformation with construct for *MtWOX2* overexpression on callus growth. (**A**,**B**). Boxplots representing the number of somatic embryos per callus (**A**) or weight (**B**) of transgenic T0 calli with *GUS* and MtWOX2 overexpression. Data were obtained from 20–22 calli for different genotypes. To assess the statistical significance of the observed differences, the Wilcoxon signed-rank test was used. (**C**,**D**). Boxplots representing the number of somatic embryos per callus (**C**) or weight (**D**) of calli obtained from R108 plants and plants containing construct for *MtWOX2* overexpression. Data were obtained from 4–7 calli for different genotypes.

On the 40th day of cultivation on the medium for regeneration, the calli were collected, and their weight and the number of embryos per callus were evaluated. Calli overexpressing *MtWOX2* (MtWOX2oe calli) did not demonstrate an increased number of somatic embryos per callus (Figure 1A). Their median weight was greater in comparison with the control calli; however, the differences in values were not statistically significant, according to the Mann–Whitney test (Figure 1B). At the same time, we observed several individual calli of a very large weight (more than 1 g), which occurred only in the MtWOX2oe genotype (Figure S3). When the calli were divided into two categories (weight greater than 1 g and less than 1 g), Fisher’s exact test confirmed that *MtWOX2* overexpression is a significant factor influencing the frequency of occurrence of calli greater than 1 g (*p*-value = 0.007389).

To check our results, we obtained two independent lines containing the construct for *MtWOX2* overexpression. T1 plants from these lines and R108 plants were used as a source of leaf explants, which were cultivated on callus formation and regeneration media, similarly to the previous experiment, but without antibiotics in the media. As a result, calli containing constructs for *MtWOX2* overexpression indeed have a significantly larger weight than R108 calli (*p*-value = 0.0061, Mann–Whitney test), whereas no significant change in the embryo number per callus was observed (Figure 1C,D). Unlike T0 calli generation, T1 calli, obtained from transgenic MtWOX2oe T1 plants, did not demonstrate increased expression of *MtWOX2* (Figure S2B). This absence of increased *MtWOX2* expression is noteworthy, considering the significant differences in weight observed between T1 MtWOX2oe calli and control R108 calli.

### 2.3. Transcriptomic Analysis of MtWOX2 Overexpressing Calli

To find out what transcriptomic changes occur in *MtWOX2* overexpressing calli, we performed transcriptomic analysis of calli obtained after transformation of leaf explants with constructs for *MtWOX2* overexpression or *GUS* overexpression (as a control).

**Figure 2:**
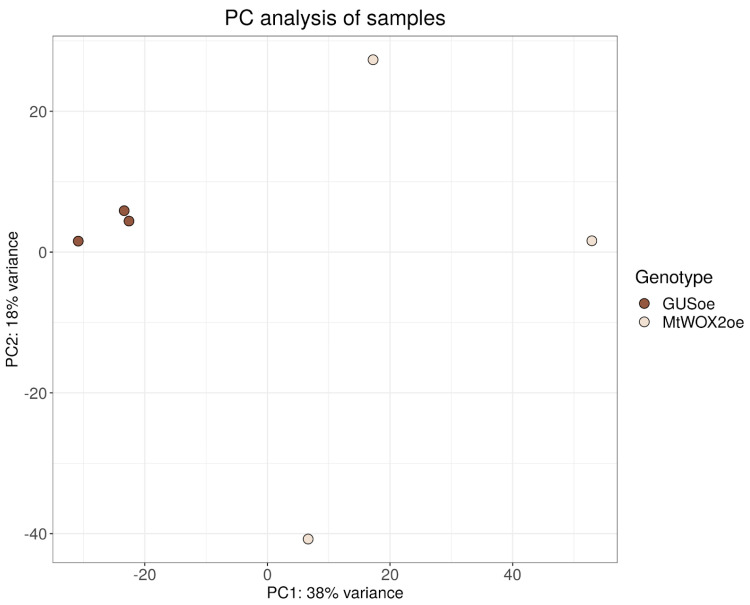
Results of principal component analysis of samples taken from *GUS* and *MtWOX2* overexpressing calli. Analysis was performed with the DeSeq2 package.

From these calli, after 43 days of cultivation (including 33 days of cultivation on the callus-inducing medium and 10 days of cultivation on a hormone-free medium), RNA was isolated, and cDNA was obtained from it and sequenced (3 biological replicates for each genotype). Differential gene expression analysis was performed using the DeSeq2 package. Principal component analysis showed that MtWOX2oe samples were more similar to each other than to control samples (Figure 2). It is worth noting that MtWOX2oe samples apparently have more differences from each other in comparison with control GUSoe group, according to the PCA. Such a high level of dispersion suggests a potential connection with the more intensive callus growth in MtWOX2oe. This phenomenon may be attributed to the typical cell structure of the callus, which has been reported to display significant heterogeneity [26].

To identify differentially expressed genes (DEGs), *p*-value and log2 fold change thresholds of 0.01 and 1.0, respectively, were adopted. For 1670 genes out of 50,803 analyzed, differential expression was detected, including 803 genes with increased expression and 894 genes with decreased expression in MtWOX2oe calli (Table S1).

**Figure 3:**
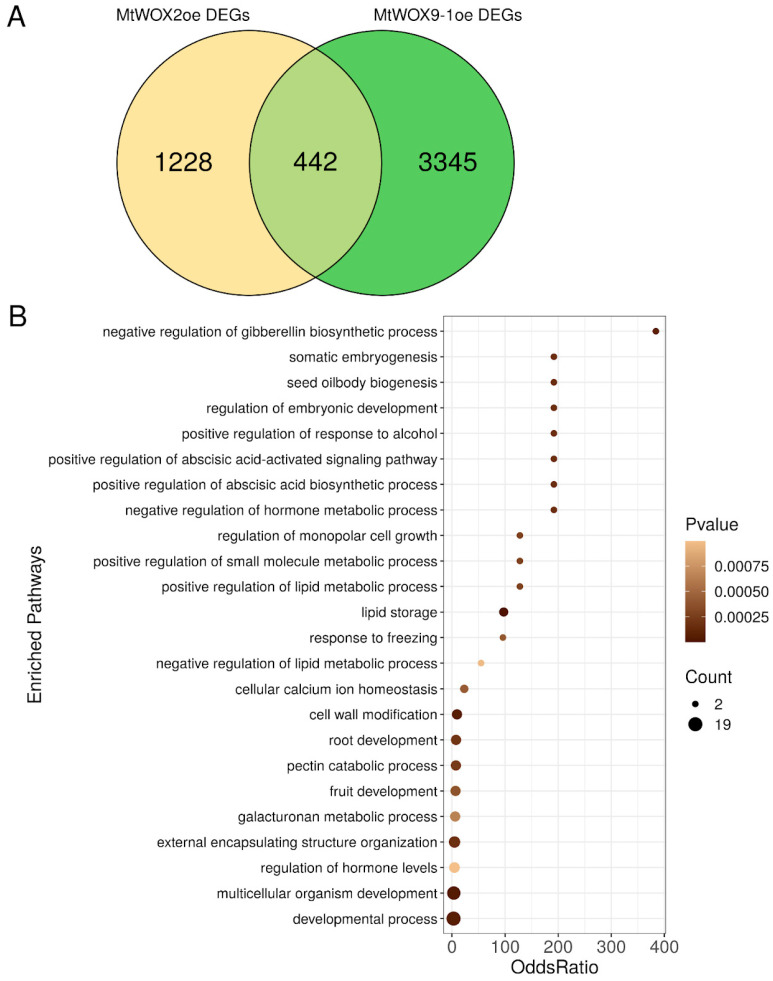
(**A**) Venn diagram representing an overlap between DEGs in MtWOX2oe calli in comparison with GUSoe calli and DEGs in MtWOX9-1oe calli in comparison with R108 calli [27]. (**B**) Overrepresented “Biological process” GO pathways in DEGs upregulated in the MtWOX9-1oe calli and downregulated in the MtWOX2oe calli.

We compared the MtWOX2oe DEGs with DEGs in calli with *MtWOX9-1* overexpression (in comparison with R108 calli) [27]. Unlike MtWOX2, which stimulated callus development according to our data, MtWOX9-1 can stimulate development of somatic embryos. MtWOX2 and MtWOX9-1 belong to the modern and intermediate clades of the WOX family, respectively. We checked if *MtWOX2* and *MtWOX9-1* overexpressing calli have some common DEGs. Indeed, 451 genes were found to show differential expression in both experiments. If the total number of genes is taken to be all genes with non-zero expression level in at least one of the analyzed samples (amounting to 34,135 genes), this value significantly deviates from the theoretically expected value (*p*-value = 4.439458 × 10^−136^, hypergeometric test). This suggests the influence of MtWOX9-1 and MtWOX2 transcription factors on significantly overlapping sets of genes (Figure 3A). Of these, 118 genes showed the same directions of change (a decrease or increase in the expression level) in the case of overexpression of *MtWOX2* and *MtWOX9-1*, and for 333 genes the directions of regulation were opposite. This distribution significantly deviates from the expected result (equal number of similarly and oppositely regulated genes) (*p*-value = 2.822 × 10^−13^, Chi-square). Therefore, we can suppose that MtWOX9-1 and MtWOX2 have at least a partly opposite impact on the callus transcriptome.

Most of the common DEGs that were oppositely regulated in *MtWOX2* and *MtWOX9-1* overexpressing calli were activated by MtWOX9-1 and repressed by MtWOX2 (216 genes out of 333). We performed GO enrichment analysis, which has shown that among these 216 genes 22 pathways are overrepresented, most of which are related to somatic and zygotic embryogenesis, morphogenesis, and seed development (Figure 3B, Table S2). Interestingly, other groups of common DEGs, including genes activated by *MtWOX2* overexpression and repressed by MtWOX9-1 overexpression as well as genes which are similarly regulated by MtWOX2 and MtWOX9-1 overexpression, had much fewer overrepresented GO pathways (8 and 1, respectively) (Figure S4).

## 3. Discussion

The search for genes which can regulate the development of tissue culture in vitro represents an important goal for biotechnology as well as for the investigation of plant development [28]. In this study, we evaluated expression patterns and possible effects on in vitro cell culture for MtWOX2, a transcription factor from the WOX family that is orthologous to the well-known embryogenesis and cell division regulator WOX2.

We did not detect specific expression of the *MtWOX2* gene during any stage of SE. These results are unexpected, because in many species *WOX2* orthologs are specifically expressed during the early stages of SE. *MtWOX2* is the only ortholog of the *WOX2* gene in the *M. truncatula* genome. Interestingly, it also does not show specific expression in the generative structures according to the MtExpress V3 dataset [29]. According to our previous studies, *STF* (*MtWOX1*), another member of the *WOX* family modern clade, has a specific expression increase during SE and it is expressed in ovules [11]. Given the redundancy between WOX2 and WOX1 in the regulation of zygotic embryo development [6], it is plausible to suggest that some *WOX2* functions during SE are carried out by the *STF* gene in *M. truncatula*.

According to our data, overexpression of *MtWOX2* led to increased callus weight, but did not have any effect on SE capacity. These data are consistent with the study, showing that ectopic *WOX2* expression is not sufficient to induce SE in *A. thaliana* [17]. Analysis of *M. truncatula* plants with *MtWOX2* loss of function will help to learn if this gene is necessary for SE.

The increased weight of calli transformed with the construct for *MtWOX2* overexpression can be caused by different factors. There is a possibility that this weight increase is related to increasing cell size or changes in the biochemical composition of cells. However, previous reports on the *WOX2* gene and its orthologs as cell division regulators suggest that such calli exhibit more intensive proliferation of non-differentiated callus cells.

Interestingly, we did not detect an increased level of *MtWOX2* expression in calli obtained from T1 plants containing constructs for *MtWOX2* overexpression, yet these calli demonstrated significantly increased weight compared to control. This may suggest some epigenetic inherited changes caused by *MtWOX2* overexpression in T0 calli, but, at the same time, allows for consideration of the negative influence of *MtWOX2* overexpression on plant development from which only transgenic plants with silenced transgene overexpression can regenerate or survive. That hypothesis is supported by the transcriptomic analysis of MtWOX2oe calli showing the repressive effect of *MtWOX2* on genes related to development and morphogenesis.

Transcriptomic analysis of calli with *MtWOX2* overexpression revealed that a significant portion of DEGs in such calli also are affected by *MtWOX9-1* overexpression. Furthermore, most of those genes were activated by *MtWOX9-1* and repressed by *MtWOX2*. This supports the data on WOX2 and WOX9 tobacco orthologs, which demonstrated their repressing and activating activities in a luciferase assay [30]. Interestingly, the category of genes activated by MtWOX9-1 and repressed by MtWOX2 was enriched with multiple GO terms related to embryogenesis and development in general, whereas other categories (genes activated by *MtWOX2* and repressed by *MtWOX9-1*, as well as genes similarly regulated by overexpression of the *MtWOX9-1* and *MtWOX2* genes) exhibited much fewer overrepresented GO pathways. That allows for the suggestion that MtWOX2 acts antagonistically to MtWOX9-1 in embryogenic calli and these two TFs promote two alternative pathways of cell development during in vitro cultivation: callus formation or somatic embryogenesis.

*MtWOX9-1* has specific expression during SE and in ovules [11]. Although there are three other genes orthologous to *WOX9* in the *M. truncatula* genome [31], none of them demonstrate significant expression levels in any *M. truncatula* tissue, according to the MtExpress V3 dataset [29]. At the same time, the *M. truncatula* genome lacks the *WOX8* ortholog [32]. It should be noted that in *Nicotiana tabacum*, which also lacks *WOX8* orthologs, *NtWOX9* together with *NtWOX2* are co-expressed in the zygote [30], suggesting that WOX9 and WOX8 orthologs can perform each other’s functions in different species. These data suggest that MtWOX9-1 carries out both WOX8 and WOX9 functions in *M. truncatula* development. *MtWOX2* transcription is stimulated by *MtWOX9-1* overexpression, according to the transcriptome analysis [27]. Our data on the antagonistic effects of *MtWOX2* and *MtWOX9-1* in the embryogenic calli suggest the existence of a negative feedback loop between these two genes, reminiscent of WOX2 and WOX8 relations in the zygotic embryos [21].

Together, our results add new information on the relationships between different *WOX* genes and can be used in biotechnology for species with poor callus formation.

## 4. Materials and Methods

### 4.1. Plant Material and Bacterial Strains

Plants of *Medicago truncatula* A17 and 2HA lines derived from the Jemalong cultivar and the R108 line derived from ecotype 108-1 were used in the study. Seeds of the A17 line were provided by colleagues from Wageningen University (The Netherlands). Seeds of the 2HA line were provided by Dr Mireille Chabaud (French National Institute for Agriculture, Food, and Environment)). Seeds of the R108 line were provided by colleagues from Samuel Roberts Institute (USA).

*Rhizobium radiobacter* (*Agrobacterium tumefaciens*) AGL1 strain and *Escherichia coli* TOP10 strain were used for plant transformation and cloning, respectively.

### 4.2. Plant Cultivation Conditions

For germination, *M. truncatula* seeds were treated with concentrated sulfuric acid (95–97%) for 10 min, then washed 10 times with sterile distilled water. Sterilized seeds were germinated on the 1% agar at 4 °C in the dark. Plants were grown in soil and under in vitro conditions at 21–24 °C, photoperiod 16 (light)/8 (dark). Terra Vita soil (Nord Pulp, St. Petersburg, Russia) mixed with vermiculite (3:1) was used for growth in growth chambers. Modified Fahraeus medium [22,33] was used for growth in sterile conditions.

Plant transformation and the obtaining of R108 line T0 calli were performed as described in [22]. For callus induction, modified PCI-4 medium [34] was used with 4 mg/L (18 μM) 2,4-D, 0.5 mg/L (2.22 μM) BAP, 250 mg/L cefotaxime and 25 mg/L hygromycin. The medium for the SE induction has the same composition as callus induction medium, but it did not contain hormones and had 12.5 mg/L hygromycin instead of 25 mg/L. The SE capacity and weight of T0 calli were assessed on day 74 after transformation (34 days of culturing on callus induction medium and 40 days of culturing on hormone-free medium).

Transgenic T0 plants from transformed calli were obtained by further cultivation of calli on the medium for the SE induction. After the formation of 1–2 true leaves, the regenerants were transferred to the germination medium-modified PCI-4 medium [34] without any hormones and antibiotics, and then, after 10–14 days, to the rooting medium (half-strength modified PCI-4 medium [34] without hormones and antibiotics). After the explants formed roots, they were transferred to the modified Fahraeus medium [22,33] for 10–14 days and then to the soil to obtain seeds.

In vitro cultivation of leaf explants from R108 and MtWOX2oe T1 transgenic plants was performed as described in [22]. Callus-inducing and SE-inducing media had the same composition as the respective media for obtaining T0 calli, but they did not contain cefotaxime and hygromycin. The SE capacity and weight of T1 calli were assessed on day 75 after transformation (40 days of culturing on callus induction medium and 35 days of culturing on hormone-free SE induction medium).

In vitro cultivation of leaf explants from A17 and 2HA lines was performed as described in [35].

### 4.3. Microorganism Cultivation Conditions

*E. coli* bacteria were grown in solid or liquid LB medium in standard cultivation conditions [36]. The transformation of *E. coli* was performed according to [37]. *A. tumefaciens* bacteria were grown in solid or liquid YEP medium (per 1 L of distilled water: 5 g NaCl, 10 g tryptone, 10 g yeast extract, 15 g agar (in case of solid medium)). Transformation of *A. tumefaciens* was performed using the freeze-thaw method [38].

### 4.4. Molecular Cloning and qPCR Analysis

For molecular cloning, the Gateway method [39] was used. The *MtWOX2* coding sequence was cloned into the pDONR207 vector which was used as a donor plasmid. For overexpression, the pMDC32 destination plasmid was used [40]. Plasmids were isolated from bacteria night cultures using the Plasmid MiniPrep kit (Evrogen, Moscow, Russia). The cds fragments were isolated from the agarose gel using the Cleanup Mini kit (Evrogen).

For qPCR analysis, total RNA was isolated from plant tissues using TRIzol reagent (ThermoFisher Scientific, Waltham, MA, USA) according to the manufacturer’s instructions. DNA was purified using the RapidOut DNA Removal Kit (ThermoFisher Scientific). cDNA was synthesized from 100–500 ng of RNA. Reverse transcription was performed with RevertAid reverse transcriptase, RiboLock RNA inhibitor (ThermoFisher Scientific), and oligo-dT18 primer in a volume of 20 μL according to the manufacturer’s instructions. The cDNA samples were diluted with deionized water up to 100 µL. A kit with Eva Green dye (R-441, Syntol, Moscow, Russia) was used for qPCR. qPCR was performed in the CFX96 Real-Time PCR Detection System (Bio-Rad, Hercules, CA, USA). The values of threshold cycles were estimated using CFX-Manager software (Bio-Rad). The *MtH3L* gene was used as a reference. The delta-delta Ct method was used for qPCR data processing [41]. Primers used in the study are listed in the Table S3. Primers for qPCR for the *MtH3L* reference gene were taken from [42].

Ugene (version 37.1) [43], SnapGene Viewer (version 5.2.1, from GSL Biotech; available at snapgene.com), Primer3 [44], 2012), and ApE (version 2.0.7) (M. Wayne Davis) were used for sequence analysis and primer design. Statistical analysis and diagram drawing were performed using RStudio (RStudio Team, 2020).

### 4.5. Transcriptome Analysis

Library preparation and sequencing were carried out using Illumina technology by the resource center of St. Petersburg State University “Development of molecular and cellular technologies”. The library was prepared using the NEBNext^®^ Ultra II Directional RNA Library Prep Kit for Illumina (New England Biolabs, Ipswich, MA, USA). Sequencing was performed on a HiSeq4000 instrument in PE151 sequencing mode.

Raw sequence files were screened using FastQC [45]. Trimming of low-quality sequences was performed with the BBDuk package belonging to the BBMap software (version 39.01) [46]. Alignment on reference genome (MtrunA17r5.0-ANR, [32]) was performed by the HISAT2 program [47], and reads were counted with the StringTie software package (version 2.2.0) [48] using the reference genome mentioned above and without de novo assembled transcripts. Differential gene expression analysis was performed using the DeSeq2 package (version 1.40.2) [49]. To identify DEGs, *p*-value and log2 fold change thresholds of 0.01 and 1.0, respectively, were adopted. The enriched Gene Ontology (‘Biological process’) groups were analyzed using the GSEAbase package (version 1.64.0).

## 5. Conclusions

The potential role of MtWOX2 as a stimulator of callus development has been demonstrated. Transcriptomic analysis reveals predominantly contrasting effects of MtWOX2 and MtWOX9-1 transcription factors on the development of the embryogenic callus.

## Data Availability

The data presented in this study are available in Tables S1 and S2.

## Supplementary Materials

The following supporting information can be downloaded at: https://www.mdpi.com/article/10.3390/plants13010102/s1, Figure S1. *MtWOX2* gene expression dynamics during in vitro cultivation of explants of embryogenic 2HA (pink) and non-embryogenic A17 (dark-red) lines. Error bars represent the standard error. Data were obtained from three biological replicates. To assess the statistical significance of the observed differences, one-way analysis of variance (one-way ANOVA) with Tukey’s post hoc test was used, with confidence level 0.95. Different lower case letters represent expression levels with statistically significant differences (*p*-value < 0.05). Figure S2. (**A**) Expression levels of *MtWOX2* in T0 calli from explants transformed with constructs for *GUS* (GUSoe) and *MtWOX2* (MtWOX2oe) overexpression. (**B**) Expression levels of *MtWOX2* in calli obtained from control R108 plants and from T1 plants containing constructs for *MtWOX2* overexpression. Error bars represent the standard error. Data were obtained from 4-7 biological repeats per genotype. To assess the statistical significance of the observed differences between different genotypes of calli, the Wilcoxon signed-rank test was used. Figure S3. (**A**,**B**) Calli obtained from explants transformed with constructs for *GUS* (**A**) and *MtWOX2* (**B**) overexpression. Scale bars are 1 cm. (**C**) Mosaic plot showing number of calli with weight exceeding 1 g (yellow) or not exceeding 1 g (brown). (**D**) Bar plot representing frequencies of T0 GUSoe and MtWOX2oe calli with different weights. Figure S4. (**A**) Overrepresented “Biological process” GO pathways in genes downregulated in MtWOX9-1oe calli and upregulated in MtWOX2oe calli. (**B**) Overrepresented “Biological process” GO pathways in DEGs with similar sign of expression change in MtWOX9-1oe calli and MtWOX2oe calli. Table S1: List of DEGs in calli with *MtWOX2* overexpression in comparison with calli with *GUS* overexpression. Table S2. DEGs corresponding to the enriched GO terms (“Biological Process”) in genes activated by *MtWOX9-1* overexpression and repressed by *MtWOX2* overexpression. Table S3: Primers used in the study.
